# Selection and validation of reference genes for quantitative real-time PCR of *Quercus mongolica Fisch*. *ex Ledeb* under abiotic stresses

**DOI:** 10.1371/journal.pone.0267126

**Published:** 2022-04-28

**Authors:** Hao Zhan, Hanzhang Liu, Tianchong Wang, Lin Liu, Wanfeng Ai, Xiujun Lu

**Affiliations:** 1 College of Horticulture, Shenyang Agricultural University, Shenyang, China; 2 College of Forestry, Shenyang Agricultural University, Shenyang, China; 3 Key Laboratory of Forest Tree Genetics, Breeding and Cultivation of Liaoning Province, Shenyang, China; Institute for Sustainable Plant Protection, C.N.R., ITALY

## Abstract

*Quercus mongolica Fisch*. *ex Ledeb* is the main species of coniferous and broadleaved mixed forests in northeast and north China, which has high ornamental, economic, and ecological value. The appropriate reference genes must be selected for quantitative real-time PCR to reveal the molecular mechanisms of stress responses and their contribution to breeding of *Q*. *mongolica*. In the present study, we chose 11 candidate reference genes (*TUA*, *CYP18*, *HIS4*, *RPS13*, *ACT97*, *TUB1*, *UBQ10*, *UBC5*, *SAND*, *PP2A*, and *SAMDC*) and used four programs (GeNorm, NormFinder, BestKeeper, and RefFinder) to assess the expression stability of the above genes in roots, stems, and leaves under five abiotic stress factors (cold, salt, drought, weak light, and heavy metal). The findings revealed that under various experimental environments, the most stable genes were different; *CYP18*, *ACT97*, and *RPS13* ranked the highest under most experimental environments. Moreover, two genes induced by stress, *CMO* and *P5CS2*, were chosen to demonstrate the reliability of the selected reference genes in various tissues under various stress conditions. Our research provides a significant basis for subsequent gene function studies of *Q*. *mongolica*.

## Introduction

Gene expression analysis is crucial for revealing the mechanisms underlying different biological processes, including signal transduction in plants, metabolic pathways, tissue development, and stress response at the molecular level [1–4]. qRT-PCR is presently an important strategy employed for investigating gene expression because of its specificity, accuracy, efficiency, and high sensitivity [5, 6]. However, qRT-PCR can be substantially affected by several factors such as the choice of the reference gene, amplification efficiency of reverse transcription PCR, and quality and purity of RNA. Hence, the appropriate reference gene must be chosen for correction and standardization to avoid errors in qRT-PCR assays.

The proper reference genes should be stably expressed under various experimental environments and in all sample tissues. Some housekeeping genes, such as glyceralde-hyde-3-phosphate dehydrogenase (*GAPDH*), actin (*ACT*), ubiquitin (*UBQ*), and beta-tubulin (*TUB*), have been widely employed as reference genes for plant qRT-PCR [7–10]. Nonetheless, increasing studies have revealed that the expression levels of these generally employed reference genes may differ under experimental environments or in various tissues and are generally applicable to specific plant species [11–13]. Moreover, the current research usually selects reference genes reported in model plants for gene expression analysis because of the lack of genome information regarding non-model plants [14]. Nevertheless, the stability of reference genes requires further verification because using the inappropriate reference gene will cause errors in the description of the expression level of the target gene [15]. Therefore, stable reference genes must be chosen according to the actual conditions.

The genus *Quercus* belongs to the Fagaceae family, containing 300 to 600 large woody deciduous and evergreen species that are distributed from tropical to temperate regions and are an important part of forests in the Northern Hemisphere [16]. Among deciduous species of the genus *Quercus*, *Quercus mongolica Fisch*. *ex Ledeb* is one of the dominant species with strong resistance in warm temperate forests and is the main species of coniferous and broadleaved mixed forests in northeast and north China [17]. *Quercus mongolica* not only has high economic value (for example, it is used to breed large-diameter timber and raise tussah, an important silk-secreting insect in China) but also plays an important role in maintaining a regional ecological balance, protecting the ecological environment, and restoring and rebuilding the ecosystem [17, 18].

*Quercus mongolica* has strong tolerance to cold and drought stress [18]; however, as a heliophilous species, the seedlings of *Q*. *mongolica* cannot grow normally under low light stress, which seriously affects the process of natural regeneration [19]. In addition, few reports have focused on the tolerance of *Q*. *mongolica* to other abiotic stresses. With the rapid development of molecular biology, the exploration of gene expression patterns has become possible, which can help in understanding the molecular mechanisms of stress response and can contribute to breeding and diversified utilization of *Q*. *mongolica*. Nonetheless, no study has reported the systematic verification and assessment of suitable reference genes of *Q*. *mongolica* under various abiotic stress environments; therefore, suitable reference genes for *Q*. *mongolica* stress-related gene expression analysis must be identified.

In this study, we investigated the expression stabilities for 11 candidate reference genes (*TUA*, *CYP18*, *HIS4*, *RPS13*, *ACT97*, *TUB1*, *UBQ10*, *UBC5*, *SAND*, *PP2A*, and *SAMDC*) in *Q*. *mongolica* leaves, stem, and roots under cold, salt, drought, weak light, and heavy metal stresses. The most stable gene for qPCR was determined using NormFinder, GeNorm, RefFinder, and BestKeeper. Finally, *CMO* and *P5CS2* from *Q*. *mongolica* were employed to verify the suitability of the candidate reference genes. The purpose of the current work was to assess the appropriate reference genes in specific experimental environments and to lay the foundation for future research on the expression of genes of *Q*. *mongolica*.

## Materials and methods

### Plant materials and stress treatments

Seeds of *Q*. *mongolica*, collected from LiaoNing Province in Northeast China, were used in this study. The seeds were sown in plastic pots filled with mixture of nutrient soil, perlite, and vermiculite (v/v/v 3:1:1) and were randomly placed in the artificial growth house of Shenyang Agricultural University (41°82′N; 123°56′E), Shenyang, China. The cultivation conditions were 16 h of light and 8 h of darkness, 400 μmol/(m^2^·s) light intensity, and the temperatures under light and dark conditions were 25°C and 20°C, respectively. The relative humidity was 60%. After 8 weeks of growth, the seedlings that were grown consistently were randomly collected for different experimental treatments. For drought and salt stress, the seedlings were watered with 2 L 20% PEG-6000 and 2 L 300 mM NaCl, respectively. For heavy metal stress, the seedlings were watered with 2 L 1.5mM CdSO_4_·8/3H_2_O solution. For cold stress, the seedlings were subjected to temperatures of 4°C in an incubator. For weak light stress, the seedlings were placed under 80 μmol/(m^2^·s) light intensity. The leaves, stems, and roots were sampled separately after 24, 48, and 72 h of each treatment. Untreated seedlings of *Q*. *mongolica* at 0 h were used as control plants. All the treatments were performed in the artificial growth house mentioned above. For different tissues, the samples of leaves, stems, and roots were collected from 40-year-old *Q*. *mongolica* at Experimental Forest Farm of Liaoning Province. The experiment was performed with three biological replicates. The samples were cleaned with purified water and immediately frozen in liquid nitrogen and stored at −80°C until further analysis.

### RNA isolation and cDNA synthesis

Total RNA was extracted from the leaf, stem and root tissues using a RNA Isolation Kit (TianGen, China) with DNase treatment to remove genomic DNA. RNA integrity was checked using 1.5% agarose gel electrophoresis, and the RNA concentration and purity were determined using a NanoDrop 2000 Spectrophotometer (NanoDrop, Thermo Scientific, Waltham, MA, USA). All the RNA samples with A260/A280 ratios = 1.8–2.2 and A260/A230 ratios > 2.0 were used for further study. cDNA synthesis was performed with 1000 ng of total RNA in a final volume of 20 μL using a cDNA synthesis kit (Monad, China) following the manufacturer’s instructions. The four-fold dilution cDNA was stored at −20 ˚C for further study.

### Candidate reference gene selection and primer design

According to the related research literature on plant reference genes [15, 20–23], we selected 11 candidate reference genes (*TUA*, *CYP18*, *HIS4*, *RPS13*, *ACT97*, *TUB1*, *UBQ10*, *UBC5*, *SAND*, *PP2A*, and *SAMDC*). We used the Arabidopsis nucleotide sequences as query sequences for BLAST in our *Q*. *mongolica* genome database (PRJNA609556) to identify candidate reference genes. The 11 reference gene sequences and homologous gene accession in The Arabidopsis Information Resource (TAIR) database were listed in S1 Table. Specific primers for the reference genes were designed using Primer premier 5.0 (Premier Biosoft International, Palo Alto, CA, USA). The parameters were as follows: the length of the primers and the amplification product size were 18–25 bp and 100–300 bp, respectively, the range of annealing temperature (Tm) was 60–70°C, and the GC was 40–60%. The primer sequences for qRT-PCR were listed in Table 1.

**Table 1:** Amplification features together with primer sequences of 11 candidate reference genes.

Gene symbol	Gene description	Gene ID	Primer sequence forward/reverse(5‘-3’)	Tm (°C)	Amplicon length (bp)	E(%)	R^2^
TUA	Tubulin-alpha	Qm017969	TGTTCTCCCGTATTGACCACAA	60.4	160	95.79	0.9938
			GCAGACTCAGCACCTACCTCCT	60.1			
CYP18	Cyclophilin 18	Qm030559	CAGTTCTTCATAACCCTGGCTCC	62	163	108.16	0.9869
			TGACCGATGTCCGTAGTATCTTCAC	62.4			
HIS4	Histone H4	Qm016260	ATCACAAAGCCAGCCATTCG	60.9	220	99.4	0.9977
			CCCCAAAACCATACAAGGTCC	60.8			
RPS13	Ribosomal protein S13	Qm015916	GATTCTAAGTTTCGGTTGATTCTGG	60.9	114	98.75	0.9997
			GCTGGCGGTGGTAGATTCATA	60.2			
ACT97	Actin 97	Qm028319	CCAAAGGCTAACCGTGAGAAAA	61.3	108	107.24	0.9844
			ACGACCACTGGCATAGAGGGAG	62.9			
TUB1	Tubulin-beta 1	Qm000743	TGGCATCCACTTTTATTGGTAACTC	61.9	133	100.83	0.9997
			TCCATCTCGTCCATCCCTTCA	62.4			
UBQ10	Ubiquitin 10	Qm014280	TGCGATTGCGTGGAGGAA	61.3	235	100.55	0.9985
			AGGCGAAGCACCAGATGAAG	60.2			
UBC5	Ubiquitin-conjugating enzyme E2 5	Qm006603	GCTTCTACTGTATCCCAACCCATC	61.2	214	118.01	0.9888
			TCTGCCTGCCACTTGCTCAT	61.4			
SAND	SAND protein family	Qm027872	AATTGCCTACCGATCCATTACC	60.1	122	106.32	0.9928
			CCAAAGTCCAGCAGGACCAC	60			
PP2A	Protein phosphatase 2A	Qm013217	GAGGTTCCACATGAAGGACCAA	61.2	278	96.12	0.9866
			TCCCCAATCTCCAGAATAGCAG	60.9			
SAMDC	S-adenosylmethionine decarboxylase	Qm002492	AATCAAAACTTGCGGGACCAC	61.5	275	96.53	0.9967
			CCATTTCGGCAGAAGCAGAGTA	62.1			
P5CS2	Delta1-pyrroline-5-carboxylate synthase 2	Qm016368	AATGACAGTTTGGCTGCTCTGC	61.9	107	107.53	0.9904
			GACTTTGGGTCACTTGGAGGG	60.8			
CMO	Choline monooxygenase	Qm002895	CAAACGCAAATCCATTCCCTC	61.9	296	111.01	0.9936
			CACGAATGTTTCCCTTTTCATCTC	62			

R^2^: regression coefficient; E:amplification efficiency; Tm: annealing temperature.

To test the specificity of the qRT-PCR primers, the amplification product of PCR for each gene was verified by 1.5% agarose gel electrophoresis and the melting curve was included after amplification. Amplification efficiency (E) and correlation coefficients (R^2^) were calculated using a standard curve formed by using a ten-fold dilution (1, 1/10, 1/100, 1/1000, and 1/10,000) of cDNA. The amplification efficiency (E) was calculated as E = [10 ^(−1/slope)^ − 1] ×100%.

### qRT-PCR analysis

The reactions were performed on a Step One Plus RT-PCR system (Applied Biosystems, USA) with TB Green Premix Ex TaqTM Ⅱ (TliRNaseH Plus) (TaKaRa, China) in accordance with the manufacturer’s instructions. Each reaction mixture (10 μL) contained 1 μL of diluted cDNA (250 ng μL^−1^), 5 μL of TB Green Ⅱ, 0.2 μL ROX Reference Dye, 0.5 μL of each primer (10 μM) and 2.8 μL of ddH_2_O. The qRT-PCR was performed under the following conditions: 95°C for 30 s, followed by 40 cycles of 95°C for 5 s, 53°C for 30 s and 72°C for 30 s, then dissolution at 95°C for 15 s, 60°C for 1 min and 95°C for 15 s. Three independent replications were performed.

### Stability analysis of candidate reference genes

Three different software programs GeNorm [24], NormFinder [25], BestKeeper [26] and the RefFinder [22] web tool were used to evaluate the stability of 11 candidate reference genes under various stress conditions.

### Validation of candidate reference genes

To validate the reliability of the reference genes from software programs analysis, two widely known abiotic stress-related genes, namely *P5CS2* and *CMO*, were selected to measure the expression stability of the candidate reference genes. The gene expression levels were normalized with the two most stable, as well as the two least stable, candidate reference genes identified from this study. Finally, we used the 2^-△△CT^ method to calculate the relative expression levels of the verified genes [12]. Statistical analyses were performed using one-way ANOVA with SPSS 21.0 software.

## Results

### PCR specificity and qRT-PCR amplification efficiency

Eleven candidate reference genes were selected for qRT-PCR analysis. For the above genes, Table 1 revealed the gene ID, the name of the genes, R^2^ and E values, and the amplicon length. We applied 1.5% Agarose gel electrophoresis to analyze the amplified products, and only one desired size band was observed; non-specific amplification or primer dimers were not observed (S1 Fig). For specific detection of PCR, after amplification, a single peak was observed in the melting curve (S2 Fig). For PCR, the value of E varied from 95.79% for *TUA* to 118.01% for *UBC5*, and for the standard curve, the correlation coefficient ranged from 0.9844 for *ACT97* to 0.9997 for *RPS13* (S3 Fig).

### Stability of expression of candidate reference genes

**Fig 1:**
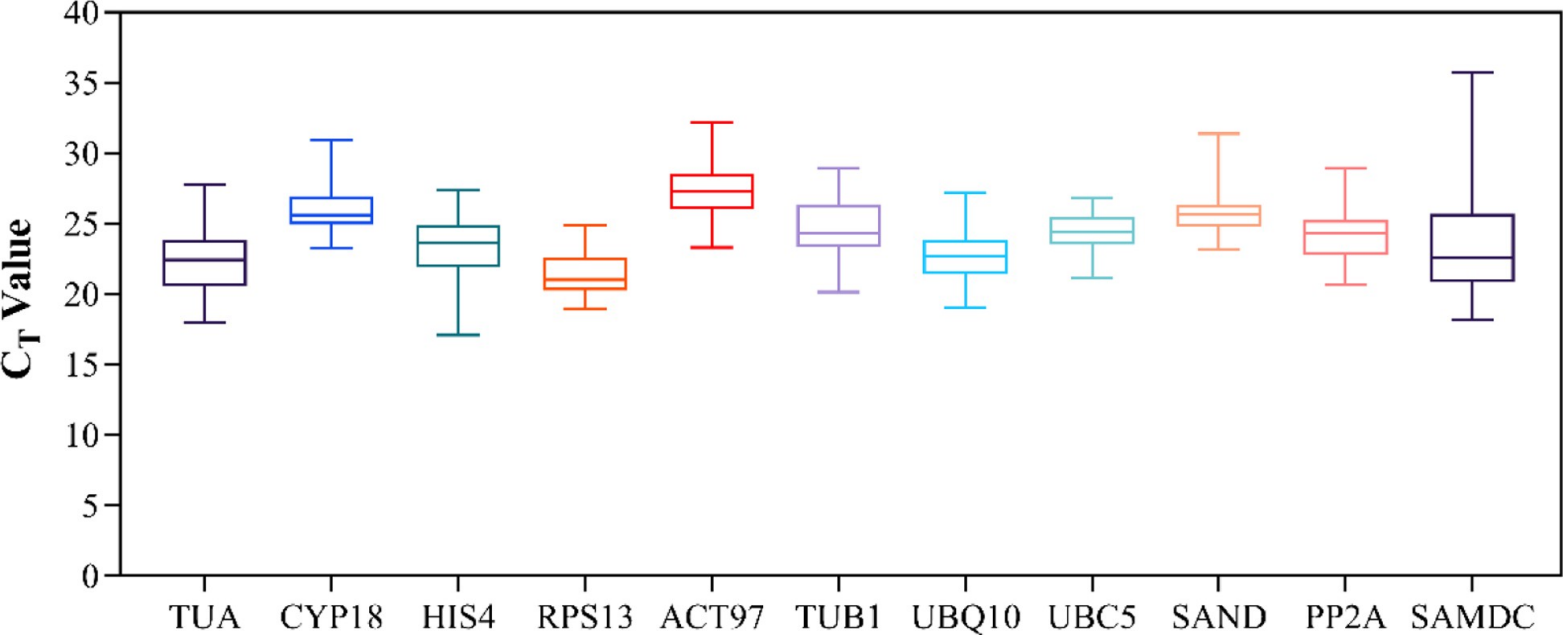
The distribution of Ct values of 11 candidate reference genes of Q. *mongolica* leaves, stem, and roots under various abiotic stress conditions. The line across the box and the lower and upper boundaries of the box are the 50th, 25th and 75th percentile, respectively. Whisker caps represents the maximum and minimum values.

The distribution of Ct values of 11 genes in all detected samples is shown in Fig 1. Overall, the mean Ct values for all samples were between 21.40 and 27.30. The Ct value in *RPS13* was the lowest (21.40), whereas that of *ACT97* was the highest (27.30).

The Ct values of all samples were generally between 17.10 and 32.17. The Ct value of *UBC5* was between 21.14 and 26.86, which means that in all samples, *UBC5* is the most stable, followed by *RPS13* and *CYP18*, with Ct values between 18.93 and 24.91 and between 23.26 and 30.96, respectively. The Ct value of *SAMDC* changed the most (between 18.15 and 31.50), which means that *SAMDC* is the most unstable reference gene in the experimental environment. Similarly, the Ct values of *HIS4* and *TUA* were between 17.10 and 27.37 and between 18.01 and 27.78, respectively, second only to *SAMDC*. The above results showed that the expression levels of the reference gene were different under various stress environments and tissues. Thus, *Q*. *mongolica* reference genes must be screened under specific environments.

### GeNorm analysis

**Fig 2:**
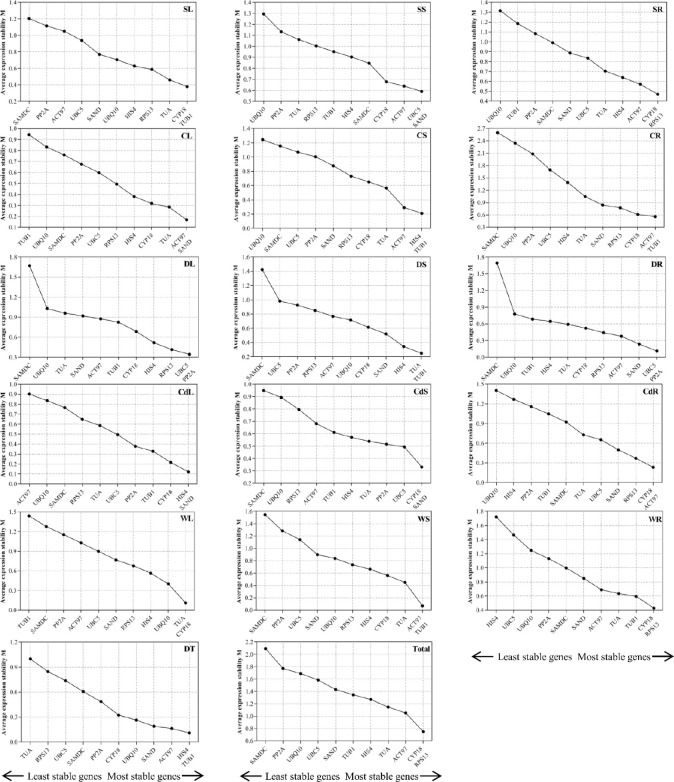
The stability measurement (M) of the expression of 11 candidate reference genes in *Q*. *mongolica* leaves, stem, and root samples under the five abiotic stress environments analyzed by GeNorm software. CR: Cold-treated roots; CS: Cold-treated stem; CL: Cold-treated leaves; SR: Salt-treated roots; SS: Salt-treated stem; SL: Salt-treated leaves; DR: Drought-treated roots; DS: Drought-treated stem; DL: Drought-treated leaves; CdR: Cd-treated roots; CdS: Cd-treated stem; CdL: Cd-treated leaves; WR: Weak light-treated roots; WS: Weak light-treated stem; WL: Weak light-treated leaves; DT: Different tissues; Total: All samples.

In the GeNorm analysis, the M values were counted to rank the mean stability of expression in 11 candidate reference genes (S2 Table and Fig 2). Previous studies have demonstrated that M values below a threshold of 1.5 indicate appropriate reference genes. A lower value of M suggests that the expression stability of the candidate reference gene is high. In the present study, the combinations of most stable reference genes in leaves, stems, and roots samples under various abiotic stresses were different: *CYP18*/*RPS13* in all samples, *HIS4*/*TUB1* in different tissues (DT), *CYP18*/*TUB1* in salt-treated leaves (SL), *SAND*/*UBC5* in salt-treated stems (SS), *CYP18*/*RPS13* in salt-treated roots (SR), *ACT97*/*SAND* in cold-treated leaves (CL), *TUB1*/*HIS4* in cold-treated stem (CS), *ACT97*/*TUB1* in cold-treated roots (CR), *PP2A*/*UBC5* in drought-treated leaves (DL) and drought-treated roots (DR), *TUB1*/*TUA* in drought-treated stem (DS), *SAND*/*HIS4* in Cd-treated leaves (CdL), *CYP18*/*SAND* in Cd-treated stem (CdS), *CYP18*/*ACT97* in Cd-treated roots (CdR), *CYP18*/*TUA* in weak light-treated leaves (WL), *ACT97*/*TUB1* in weak light-treated stem (WS), and *CYP18*/*RPS13* in weak light-treated roots (WR).

**Fig 3:**
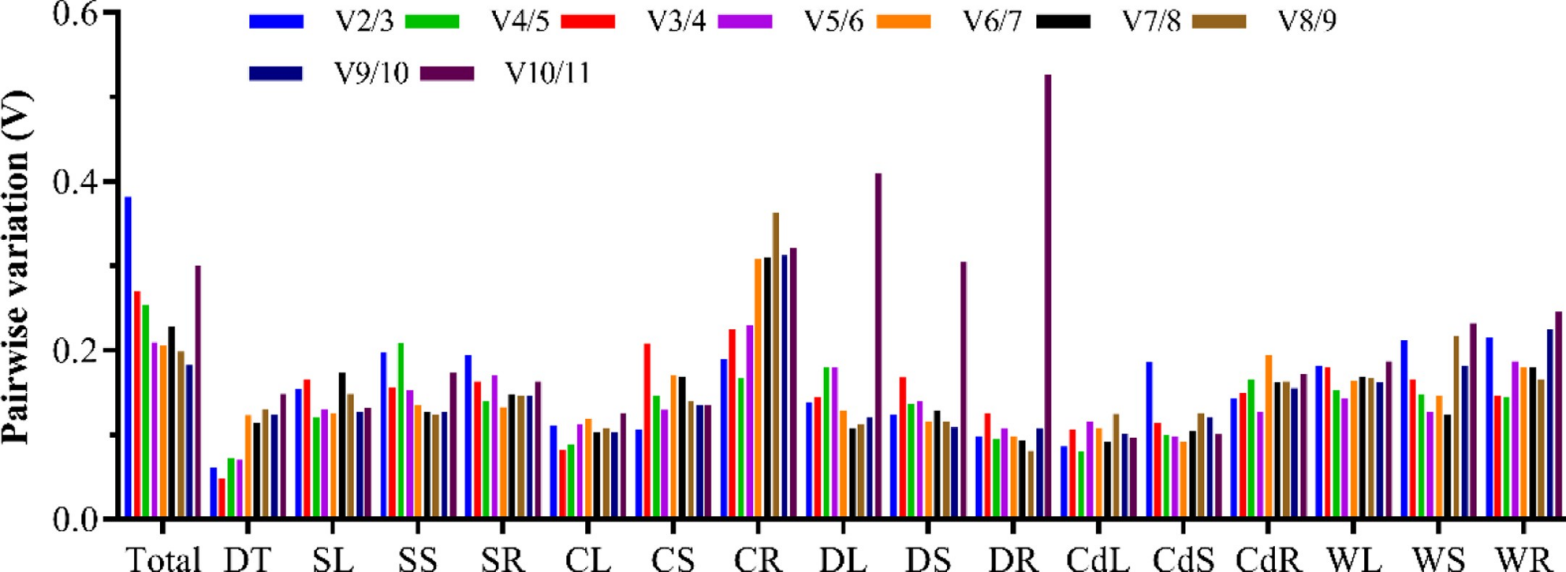
Pairwise variation (V) analysis of the 11 candidate reference genes using GeNorm under various experimental conditions. CR: Cold-treated roots; CS: Cold-treated stem; CL: Cold-treated leaves; SR: Salt-treated roots; SS: Salt-treated stem; SL: Salt-treated leaves; DR: Drought-treated roots; DS: Drought-treated stem; DL: Drought-treated leaves; CdR: Cd-treated roots; CdS: Cd-treated stem; CdL: Cd-treated leaves; WR: Weak light-treated roots; WS: Weak light-treated stem; WL: Weak light-treated leaves; DT: Different tissues; Total: All samples.

The pairwise variation (V) between the normalization factors counted through the GeNorm algorithm can also be applied to determine the optimal number of reference genes required for an accurate normalization. The cutoff of V_n/n+1_ less than 0.15 generally suggests that no additional reference genes are required for standardization. Nonetheless, a value of 0.2 was also regarded as acceptable [27]. In our study, the values of V_2/3_ in DT (0.062), DL (0.138), CS (0.106), CL (0.111), DR (0.099), DS (0.124), CdR (0.143), and CdL (0.086) samples were lower than 0.15, and the values of V_2/3_ in SR (0.194), SS (0.198), SL (0.155), CdS (0.187), CR (0.189), and WL (0.182) samples were lower than 0.2, suggesting that under these treatments, the two reference genes were sufficient to achieve exact normalization (S3 Table and Fig 3). The V_3/4_ values for WS (0.165) and WR (0.146) samples were lower than 0.2, indicating that three reference genes could be used for accurate standardization. The value of V_8/9_ in total samples (0.199) was lower than 0.2, suggesting that eight reference genes should be employed for accurate normalization. Certainly, the V_n/n+1_ value of total samples was merely a recommendation and samples could be analyzed separately based on the concerned tissue and/or stress in normal set up. Nonetheless, it has been reported that the use of multiple reference genes may increase the complexity and instability of experiments [28], revealing that accurate standardization can be completed by one reference gene [29, 30]. Thus, the optimal reference gene must be chosen based on various experimental environments.

### NormFinder analysis

NormFinder assessed the stability of the reference gene through SV; a relatively low SV value generally exhibits higher stability. NormFinder counted the SV of 11 reference genes, and the results are shown in Table 2. The expression stability of *RPS13* was the highest in the total and CR samples. *UBQ10* was the most stable in SL samples, whereas the most stable gene in SS samples was *ACT97*. In addition, *TUA* revealed excellent gene expression stability in the CS and SR samples, and *TUB1*, *HIS4*, and *SAND* revealed excellent expression stability in the WR, WS, and DS samples, respectively. *PP2A* displayed the highest gene expression stability in the CdL and DR samples. Simultaneously, *CYP18* showed the highest gene expression stability in the WL, DL, CL, CdR, CdS, DT and all samples.

**Table 2:** Expression stability for 11 candidate reference genes counted through NormFinder.

**Rank**	**Total**	**DT**	**SL**	**SS**	**SR**	**CL**	**CS**	**CR**	**DL**
	**Gene(SV)**	**Gene(SV)**	**Gene(SV)**	**Gene(SV)**	**Gene(SV)**	**Gene(SV)**	**Gene(SV)**	**Gene(SV)**	**Gene(SV)**
1	RPS13(0.761)	CYP18(0.135)	UBQ10(0.134)	ACT97(0.307)	TUA(0.191)	CYP18(0.099)	TUA(0.153)	RPS13(0.396)	CYP18(0.184)
2	CYP18(0.818)	ACT97(0.136)	SAND(0.148)	UBC5(0.571)	ACT97(0.377)	TUA(0.161)	CYP18(0.153)	ACT97(0.906)	SAND(0.309)
3	ACT97(0.898)	HIS4(0.156)	RPS13(0.204)	CYP18(0.578)	SAND(0.590)	SAND(0.259)	HIS4(0.697)	CYP18(0.982)	TUB1(0.418)
4	SAND(0.933)	TUB1(0.183)	HIS4(0.841)	SAND(0.610)	CYP18(0.606)	ACT97(0.461)	SAND(0.716)	TUB1(1.463)	HIS4(0.565)
5	TUA(1.298)	UBQ10(0.33)	TUA(0.908)	SAMDC(0.762)	UBC5(0.615)	HIS4(0.498)	TUB1(0.749)	SAND(1.517)	RPS13(0.582)
6	HIS4(1.445)	SAND(0.428)	ACT97(1.030)	RPS13(0.868)	HIS4(0.893)	PP2A(0.578)	ACT97(1.050)	UBC5(1.786)	ACT97(0.681)
7	TUB1(1.501)	UBC5(0.834)	CYP18(1.065)	HIS4(0.896)	RPS13(0.945)	UBC5(0.681)	RPS13(1.091)	HIS4(2.212)	TUA(0.899)
8	UBC5(1.546)	RPS13(1.037)	TUB1(1.100)	TUB1(1.102)	SAMDC(1.109)	UBQ10(0.861)	PP2A(1.114)	PP2A(2.483)	PP2A(1.061)
9	UBQ10(1.561)	SAMDC(1.115)	UBC5(1.108)	PP2A(1.120)	PP2A(1.223)	RPS13(0.956)	UBC5(1.188)	TUA(2.516)	UBC5(1.133)
10	PP2A(1.771)	PP2A(1.162)	PP2A(1.155)	TUA(1.304)	TUB1(1.580)	SAMDC(1.058)	SAMDC(1.359)	UBQ10(2.696)	UBQ10(1.306)
11	SAMDC(3.281)	TUA(1.624)	SAMDC(1.422)	UBQ10(1.886)	UBQ10(1.770)	TUB1(1.361)	UBQ10(1.468)	SAMDC(3.493)	SAMDC(4.499)
**Rank**	**DS**	**DR**	**CdL**	**CdS**	**CdR**	**WL**	**WS**	**WR**	
	**Gene(SV)**	**Gene(SV)**	**Gene(SV)**	**Gene(SV)**	**Gene(SV)**	**Gene(SV)**	**Gene(SV)**	**Gene(SV)**	
1	SAND(0.322)	PP2A(0.055)	PP2A(0.227)	CYP18(0.053)	CYP18(0.116)	CYP18(0.055)	HIS4(0.172)	TUB1(0.067)	
2	UBQ10(0.396)	UBC5(0.055)	UBC5(0.266)	SAND(0.165)	ACT97(0.116)	TUA(0.055)	CYP18(0.459)	RPS13(0.214)	
3	TUB1(0.439)	SAND(0.122)	CYP18(0.333)	TUB1(0.449)	RPS13(0.188)	HIS4(0.466)	UBQ10(0.471)	CYP18(0.582)	
4	CYP18(0.536)	RPS13(0.154)	SAND(0.543)	UBC5(0.575)	TUA(0.587)	UBQ10(0.517)	TUA(0.512)	SAND(0.710)	
5	TUA(0.558)	ACT97(0.221)	HIS4(0.630)	PP2A(0.605)	UBC5(0.637)	SAND(0.577)	SAND(0.656)	TUA(0.769)	
6	HIS4(0.559)	CYP18(0.539)	TUA(0.706)	ACT97(0.631)	SAND(0.670)	RPS13(0.841)	RPS13(1.054)	SAMDC(0.887)	
7	ACT97(0.681)	HIS4(0.760)	TUB1(0.713)	TUA(0.781)	PP2A(1.378)	UBC5(1.130)	TUB1(1.259)	ACT97(0.976)	
8	PP2A(1.004)	TUA(0.877)	RPS13(0.745)	RPS13(0.854)	SAMDC(1.396)	ACT97(1.374)	ACT97(1.296)	PP2A(1.437)	
9	RPS13(1.132)	TUB1(1.000)	UBQ10(0.904)	HIS4(1.018)	TUB1(1.430)	PP2A(1.426)	UBC5(1.570)	UBQ10(1.732)	
10	UBC5(1.169)	UBQ10(1.273)	SAMDC(0.913)	UBQ10(1.070)	HIS4(1.594)	SAMDC(1.766)	PP2A(1.651)	UBC5(2.236)	
11	SAMDC(3.342)	SAMDC(5.796)	ACT97(1.045)	SAMDC(1.084)	UBQ10(1.864)	TUB1(2.039)	SAMDC(2.531)	HIS4(2.680)	

SV: Stability value.

### BestKeeper analysis

The genes with the lowest SD and CV were the most stable reference genes identified via BestKeeper; relatively low values of SD (less than 1) are generally regarded to be within an acceptable variation range. Based on the CV ± SD value produced by BestKeeper (Table 3), *SAND* was the most stable in WR, SL, and all samples. *ACT97* seems to be the most appropriate reference gene in the CL, SR, and SS samples. *CYP18* was the most stable in the DS and CS samples. *HIS4* ranked the highest in the DR, WS, and WL samples. In the CdL and DL samples, *UBC5* ranked the highest. *TUA*, *UBQ10*, *TUB1*, and *PP2A* were the optimal reference genes in the DT, CdR, CdS, and CR samples, respectively.

**Table 3:** Expression stability of 11 candidate reference genes counted via BestKeeper.

**Rank**	**Total**	**DT**	**SL**	**SS**	**SR**	**CL**	**CS**	**CR**	**DL**
	**Gene(CV±SD)**	**Gene(CV±SD)**	**Gene(CV±SD)**	**Gene(CV±SD)**	**Gene(CV±SD)**	**Gene(CV±SD)**	**Gene(CV±SD)**	**Gene(CV±SD)**	**Gene(CV±SD)**
1	SAND(4.12±1.07)	TUA(2.14±0.52)	SAND(0.95±0.23)	ACT97(1.5±0.41)	ACT97(1.39±0.39)	ACT97(1.07±0.3)	CYP18(1±0.25)	PP2A(1.87±0.45)	UBC5(3.16±0.76)
2	UBC5(4.66±1.13)	RPS13(3.32±0.79)	UBQ10(1.25±0.27)	UBC5(2.47±0.56)	CYP18(2.43±0.68)	TUA(1.26±0.28)	TUA(1.54±0.31)	UBC5(5.23±1.23)	PP2A(4.02±0.93)
3	CYP18(4.72±1.23)	UBC5(3.84±0.98)	RPS13(1.71±0.37)	RPS13(2.82±0.59)	UBC(2.81±0.67)	HIS4(1.48±0.34)	HIS4(2±0.42)	UBQ10(5.58±1.29)	SAND(4.38±1.13)
4	ACT97(5.37±1.47)	CYP18(4.79±1.33)	ACT97(2.61±0.69)	PP2A(2.88±0.63)	HIS4(3.29±0.85)	CYP18(1.53±0.39)	TUB1(2.18±0.51)	SAMDC(5.82±1.2)	RPS13(4.45±0.95)
5	PP2A(5.43±1.31)	ACT97(5.16±1.54)	HIS4(2.68±0.65)	SAMDC(2.9±0.65)	SAND(3.31±0.86)	SAND(1.62±0.41)	SAND(2.56±0.65)	CYP18(7.44±2)	HIS4(4.73±1.14)
6	UBQ10(5.59±1.26)	SAND(5.91±1.67)	PP2A(3±0.66)	SAND(3.19±0.78)	RPS13(3.42±0.78)	RPS13(2.16±0.45)	ACT97(2.57±0.66)	RPS13(7.48±1.62)	CYP18(6.1±1.57)
7	RPS13(5.65±1.21)	HIS4(6.03±1.49)	CYP18(3.21±0.84)	CYP18(3.39±0.86)	UBQ10(4.35±1.01)	UBC5(2.93±0.73)	RPS13(2.79±0.57)	ACT97(7.97±2.17)	ACT97(6.52±1.84)
8	HIS4(6.98±1.62)	SAMDC(6.12±1.51)	TUA(3.34±0.77)	HIS4(3.66±0.85)	TUA(4.39±1.02)	PP2A(3.32±0.83)	PP2A(3.7±0.89)	SAND(8.18±2.21)	TUB1(7.1±1.8)
9	TUB1(7.25±1.79)	UBQ10(6.2±1.54)	UBC5(3.44±0.82)	UBQ10(4.01±0.83)	PP2A(4.58±1.08)	UBQ10(4.01±0.92)	UBC5(3.82±0.95)	TUB1(9.82±2.31)	TUA(8.46±1.98)
10	TUA(7.49±1.67)	TUB1(6.25±1.54)	TUB1(3.5±0.89)	TUB1(4.8±1.19)	TUB1(5.67±1.51)	SAMDC(4.13±0.9)	SAMDC(4.65±0.97)	TUA(13.23±2.91)	UBQ10(9.76±2.13)
11	SAMDC(10.79±2.51)	PP2A(7.88±2.04)	SAMDC(4.27±0.86)	TUA(5.4±1.19)	SAMDC(6.56±1.62)	TUB1(4.89±1.28)	UBQ10(4.97±1.13)	HIS4(14.21±3.1)	SAMDC(14.56±3.82)
**Rank**	**DS**	**DR**	**CdL**	**CdS**	**CdR**	**WL**	**WS**	**WR**	
	**Gene(CV±SD)**	**Gene(CV±SD)**	**Gene(CV±SD)**	**Gene(CV±SD)**	**Gene(CV±SD)**	**Gene(CV±SD)**	**Gene(CV±SD)**	**Gene(CV±SD)**	
1	CYP18(0.9±0.22)	HIS4(2.02±0.5)	UBC5(1.86±0.46)	TUB1(1.67±0.4)	UBQ10(1.21±0.29)	HIS4(0.73±0.17)	HIS4(2.01±0.43)	SAND(0.97±0.25)	
2	RPS13(1.66±0.35)	CYP18(2.11±0.57)	RPS13(2.82±0.62)	SAND(1.92±0.48)	PP2A(2.08±0.52)	SAND(1.88±0.48)	CYP18(2.75±0.68)	PP2A(2.83±0.7)	
3	UBQ10(1.75±0.39)	ACT97(2.31±0.64)	SAMDC(3.18±0.7)	UBC5(1.93±0.46)	SAND(3.14±0.84)	CYP18(2.37±0.59)	UBQ10(2.78±0.61)	RPS13(2.93±0.59)	
4	ACT97(1.77±0.47)	UBQ10(3.03±0.71)	SAND(3.25±0.84)	CYP18(2.24±0.57)	CYP18(3.16±0.85)	UBC5(2.73±0.68)	TUA(3.55±0.7)	CYP18(3.36±0.86)	
5	SAND(2.17±0.53)	RPS13(3.76±0.82)	CYP18(3.27±0.85)	PP2A(2.48±0.58)	ACT97(3.17±0.88)	TUA(2.91±0.65)	SAND(3.64±0.91)	SAMDC(3.88±0.85)	
6	TUB1(2.91±0.73)	SAND(3.87±1.03)	PP2A(3.27±0.8)	ACT97(3.04±0.82)	RPS13(5.05±1.11)	RPS13(3.8±0.78)	RPS13(3.73±0.76)	TUB1(4.25±0.94)	
7	HIS4(2.95±0.67)	TUB1(3.96±0.96)	UBQ10(3.79±0.84)	RPS13(3.42±0.74)	UBC5(5.22±1.23)	PP2A(4.03±1.02)	UBC5(4.13±1.03)	UBC5(4.79±1.14)	
8	PP2A(3.04±0.67)	PP2A(3.97±0.97)	HIS4(3.84±0.94)	HIS4(3.5±0.81)	TUA(7.1±1.61)	ACT97(4.62±1.26)	ACT97(4.31±1.1)	ACT97(5.6±1.44)	
9	UBC5(3.26±0.75)	UBC5(4.01±0.96)	TUB1(4.19±1.04)	TUA(3.68±0.79)	TUB1(8.02±2)	UBQ10(4.74±1.05)	PP2A(4.36±1.06)	UBQ10(6.36±1.49)	
10	TUA(3.58±0.76)	TUA(5.08±1.14)	ACT97(4.23±1.22)	UBQ10(3.68±0.78)	HIS4(8.19±1.95)	SAMDC(5.69±1.28)	TUB1(4.65±1.06)	TUA(6.42±1.28)	
11	SAMDC(8.97±2.32)	SAMDC(14.81±4.08)	TUA(4.59±1.11)	SAMDC(4.08±0.84)	SAMDC(9.32±2.23)	TUB1(8.09±2)	SAMDC(6.77±1.47)	HIS4(12.23±2.55)	

CV: Coefficient of variation; SD: Standard deviation.

### RefFinder analysis

Finally, we used the RefFinder to generate an overall ranking of candidate genes. RefFinder is an online tool, which integrates the algorithms of GeNorm, NormFinder, and Bestkeeper. RefFinder assigns an appropriate weight to each gene and calculates the geometric mean of their weights for the overall final ranking based on the rankings from each program, and the smaller the value, the more stable the gene (Table 4). For the WR, WS, and total samples, two top-ranked genes were identified using the RefFinder program in accordance with the samples identified through GeNorm. Among all the samples, *RPS13* and *CYP18* were the most appropriate reference genes. *ACT97* and *HIS4* were the most stable reference genes in DT samples. *SAND* and *UBQ10* were the most stable in SL samples, while *ACT97* together with *CYP18* or *UBC5* were the most stable in the SR and SS samples, respectively. The most stable reference genes under cold stress were *CYP18* and *TUA* in the CS and CL samples, while *ACT97* and *RPS13* were the most appropriate for the CR samples. Under drought stress, *CYP18* together with *UBC5* or *SAND* were the most stable genes in the DL and DS samples, respectively. *UBC5* and *PP2A* were the most stable genes in the DR samples. Under heavy metal stress, *PP2A* and *UBC5* were the most suitable in the CdL samples, and *CYP18* together with *ACT97* or *SAND* were determined the most stable in the CdR and CdS samples, respectively. Under weak light stress, *CYP18* together with *TUA* or *HIS4* were stably expressed in WL and WS samples, respectively; however, *RPS13* and *TUB1* were stably expressed in WR samples.

**Table 4:** Eleven candidate reference genes ranked under various conditions using RefFinder.

**Rank**	**Total**	**DT**	**SL**	**SS**	**SR**	**CL**	**CS**	**CR**	**DL**
	**Gene(GRV)**	**Gene(GRV)**	**Gene(GRV)**	**Gene(GRV)**	**Gene(GRV)**	**Gene(GRV)**	**Gene(GRV)**	**Gene(GRV)**	**Gene(GRV)**
1	RPS13(1.32)	ACT97(2.55)	UBQ10(1.86)	ACT97(1.32)	ACT97(1.86)	TUA(1.86)	CYP18(1.78)	RPS13(2.11)	CYP18(2.34)
2	CYP18(2)	HIS4(2.59)	SAND(2.55)	UBC5(1.86)	CYP18(2.45)	CYP18(2)	TUA(2)	ACT97(2.55)	UBC5(3)
3	SAND(3.25)	TUB1(2.83)	RPS13(2.91)	SAND(3.13)	TUA(2.51)	ACT97(2.38)	HIS4(2.28)	CYP18(3.22)	PP2A(3.36)
4	ACT97(3.71)	CYP18(3.31)	HIS4(4.23)	CYP18(3.83)	RPS13(3.74)	SAND(2.59)	TUB1(2.99)	TUB1(3.46)	HIS4(3.56)
5	TUA(5.48)	UBQ10(5.48)	CYP18(4.41)	SAMDC(5)	UBC5(4.16)	HIS4(4.4)	ACT97(5.24)	PP2A(5.05)	RPS13(3.66)
6	UBC5(5.66)	RPS13(5.98)	TUA(4.79)	RPS13(5.63)	SAND(4.74)	PP2A(6.93)	SAND(5.38)	UBC5(5.42)	TUB1(4.41)
7	HIS4(6.16)	UBC5(6.03)	TUB1(4.98)	HIS4(6.7)	HIS4(5.18)	UBC5(7)	RPS13(6.59)	SAND(5.62)	SAND(4.6)
8	TUB1(7.36)	TUA(6.04)	ACT97(7.14)	PP2A(7.54)	SAMDC(8.66)	RPS13(7.35)	PP2A(7.74)	SAMDC(7.18)	ACT97(6.4)
9	UBQ10(7.77)	SAND(6.16)	PP2A(8.19)	TUB1(8.18)	PP2A(9)	UBQ1(8.94)	UBC5(8.74)	TUA(7.84)	TUA(7.64)
10	PP2A(8.8)	SAMDC(8.11)	UBC5(8.71)	UBQ10(9.82)	UBQ10(9.82)	SAMDC(9.49)	SAMDC(10)	UBQ10(7.95)	UBQ10(10)
11	SAMDC(11)	PP2A(9.12)	SAMDC(10.74)	TUA(9.97)	TUB1(10)	TUB1(11)	UBQ10(11)	HIS4(8.1)	SAMDC(11)
**Rank**	**DS**	**DR**	**CdL**	**CdS**	**CdR**	**WL**	**WS**	**WR**	
	**Gene(GRV)**	**Gene(GRV)**	**Gene(GRV)**	**Gene(GRV)**	**Gene(GRV)**	**Gene(GRV)**	**Gene(GRV)**	**Gene(GRV)**	
1	SAND(2.11)	PP2A(2.21)	UBC5(2.45)	CYP18(1.41)	CYP18(1.68)	CYP18(1.32)	HIS4(1.97)	RPS13(1.41)	
2	CYP18(2.51)	UBC5(2.89)	PP2A(2.51)	SAND(1.86)	ACT97(2.34)	TUA(2)	CYP18(2.21)	TUB1(2.45)	
3	UBQ10(3.22)	RPS13(3.16)	CYP18(2.82)	TUB1(2.82)	RPS13(2.71)	HIS4(2.45)	TUA(3.13)	CYP18(2.59)	
4	TUB1(3.31)	ACT97(3.31)	SAND(3.13)	UBC5(3.31)	SAND(4.36)	SAND(3.94)	UBQ10(3.6)	SAND(3.31)	
5	TUA(4.16)	CYP18(4.12)	HIS4(3.76)	PP2A(4.47)	TUA(5.26)	UBQ10(4.68)	TUB1(4.58)	TUA(5.03)	
6	HIS4(4.56)	HIS4(4.45)	RPS13(5.66)	TUA(6.65)	PP2A(5.45)	RPS13(5.73)	ACT97(5.03)	SAMDC(5.86)	
7	RPS13(6)	SAND(4.7)	TUB1(6.24)	ACT97(7.33)	UBC5(5.69)	UBC5(6.44)	RPS13(5.73)	PP2A(6.26)	
8	ACT97(6.09)	UBQ10(7.95)	SAMDC(7.21)	RPS13(7.67)	UBQ10(6.04)	ACT97(8.24)	SAND(5.89)	ACT97(6.59)	
9	PP2A(7.97)	TUB1(8.13)	TUA(7.36)	HIS4(8.13)	SAMDC(8.38)	PP2A(8.45)	UBC5(8.45)	UBC5(9.15)	
10	UBC5(9.74)	TUA(8.18)	UBQ10(7.98)	UBQ10(9.15)	TUB1(8.97)	SAMDC(10)	PP2A(9.46)	UBQ10(9.24)	
11	SAMDC(11)	SAMDC(11)	ACT97(11)	SAMDC(11)	HIS4(9.74)	TUB1(11)	SAMDC(11)	HIS4(11)	

GRV: geomean of ranking value.

### Validation of the candidate reference genes

**Fig 4:**
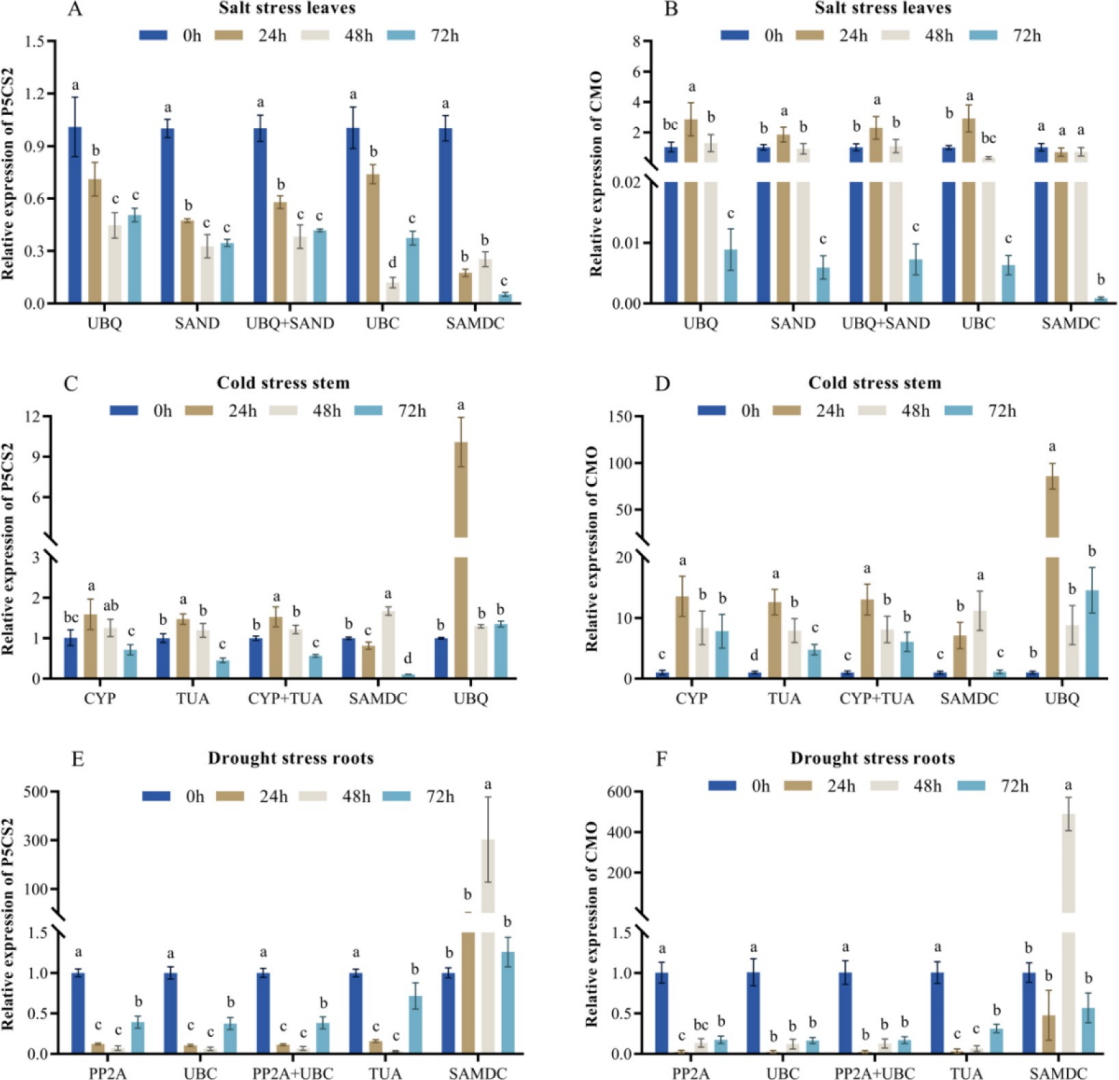
Relative gene expression levels of utilizing the chosen reference genes. The outcomes were normalized through two of the most unstable reference genes together with two most stable genes (in combination or alone) following stress treatment after 0 h, 24 h, 48 h, and 72 h. (A) The relative expression levels of *P5CS2* in SL samples. (B) The relative expression levels of *CMO* in SL samples. (C) The relative expression levels of *P5CS2* in CS samples. (D) The relative expression levels of *CMO* in CS samples. (E) *P5CS2* and *CMO* expression levels in DR samples. (F) *P5CS2* and *CMO* expression levels in DR samples. The error bar represents the standard deviation of three bio-replicates. Different letters indicate that the expression of the reference gene expression in each condition was significantly different (P < 0.05, Duncan’s test).

To verify the expression stability of the selected reference genes using the RefFinder software, *CMO* and *P5CS2* gene expression levels were quantified by employing the two most unstable genes or the two most stable genes (in combination or alone) in the RefFinder ranking (Fig 4). In the SL samples, when the most stable gene and its combination were used for normalization, the *P5CS2* expression trend was V-shaped between 0 and 72 h, and the expression reached the lowest point at 48 h; when standardized by the most unstable gene, the *P5CS2* expression trend first decreased between 0 and 72 h, then increased, and then decreased again, whereas when *UBQ10*, *SAND*, and *UBQ10* + *SAND* were used to calibrate the expression level of *CMO*, the value first increased and then decreased from 0 h to 48 h. When *SAMDC* was employed as a reference gene, the expression level of *CMO* remained stable between 0 h and 48 h and greatly decreased at 72 h. In the CS samples, when *CYP18* or *TUA* together with their combinations were applied as the reference genes, the expression trends of *CMO* and *P5CS2* exhibited an inverted V shape between 0 and 72 h, and there was a peak at 24 h. However, when the least stable gene *UBQ10* was utilized as a reference gene, the pattern of expression in *CMO* and *P5CS2* were completely different, and the relative expression levels of *P5CS2* and *CMO* were abnormally increased at 24 h. In the DR samples, when *PP2A* and *UBC5* were used as reference genes, the expression of *CMO* and *P5CS2* first declined and then increased; however, the expression pattern evidently changed when it was normalized through the least stable gene of *SAMDC*, and the value increased significantly at 48 h.

## Discussion

Quantitative determination of expression levels of major gene through qRT-PCR is an important method to understand gene function and reveal the response mechanism of plants under adversities [21, 22]. The accuracy of qRT-PCR is measured based on the choice of appropriate reference genes [31]. Ideally, the expression levels of reference genes should be stable under various experimental conditions [32], but such genes are virtually absent [33, 34]. Thus, the most appropriate qRT-PCR reference genes must be selected for specific experimental environments. The purpose of the current study was to identify the optimal reference genes for adoption in analyses of gene expression in *Q*. *mongolica* under various abiotic stress conditions. We evaluated 11 potential reference genes in *Q*. *mongolica*. According to the results, the most stable reference genes were not consistent across various experimental environments. For instance, *RPS13* and *ACT97* performed optimally in cold-treated roots, while *PP2A* and *UBC5* were more suitable in drought-treated roots.

In our research, three programs with different algorithms, BestKeeper, NormFinder, and GeNorm, were applied to assess the expression stability of candidate genes. GeNorm and NormFinder calculate the gene expression stability and then sort the candiate reference genes by SV, whereas BestKeeper calculates the results based on the SD and CV of the Ct value [24–26]. Because of the various algorithms employed in the three software packages, they have distinct rankings for identical experimental datasets [12], despite the fact that the analysis outcomes of NormFinder and GeNorm in this study hardly changed. For instance, the analysis of BestKeeper and GeNorm suggested that *PP2A* and *UBC5* were the two optimal reference genes in drought-treated leaves of *Q*. *mongolica*, but NormFinder indicated that *SAND* and *CYP18* were optimal. Furthermore, the analysis of BestKeeper suggested that *TUA* was the optimal reference gene in different tissues of *Q*. *mongolica*, but NormFinder and GeNorm indicated that the stability of *TUA* was worst. To avoid biased outcomes due to the different software programs, a fourth algorithm (RefFinder) was used to analyze the reference genes. RefFinder was applied to integrate and create comprehensive candidate reference gene rankings in accordance with the geometric average of each gene weight counted using each program [35]. Many researchers also employ RankAggreg to count the ultimate ranking [8, 11, 12]. RankAggreg applies a genetic algorithm or cross-entropy Monte Carlo algorithm to calculate the ranking of reference genes [36]. The above two tools exert a crucial effect in integrating the reference gene screening results acquired from other software.

In the present study, 11 genes that are generally applied as candidate reference genes for numerous plant species were assessed. However, the outcomes of reference gene selection are not constant in various species [11, 37], tissues [38, 39], and abiotic stress environments [14, 27]. For example, our results suggested that *UBQ10* was the most unstable in cold-treated stems, while excellent stability was determined in salt-treated leaves. Similarly, *TUA* was the most stable in cold-treated leaves, but it was unstable in salt-treated stems; *ACT97* showed excellent stability in salt-treated stems and roots but was the most unstable reference gene in the Cd-treated leaves. These results suggest that reference genes exhibit tissue specificity under certain conditions [40]. This can be explained partly through the functional diversity of reference genes; the above genes are related to the components of the cytoskeleton and participate in other cellular processes under stress environments [11]. In addition, under cold stress, *UBC5* revealed excellent stability in *Q*. *mongolica* leaves in the current study, but it was the most unstable reference gene under various abiotic environments in *Petroselinum crispum* [41]; *SAND* is the most stable reference gene in *Q*. *mongolica* stems under drought stress, but its stability in *Festuca arundinacea* leaves is poor under salt stress [7]. Similarly, *SAMDC* and *HIS4* were the least stable reference genes under most studied abiotic stress conditions in *Q*. *mongolica*, but *SAMDC* and *HIS4* showed excellent stability in drought-stressed leaves and salt-stressed roots of *Trifolium repens L*. [22] and drought-stressed leaves of *Eucommia ulmoides* [20], respectively. Moreover, *ACT97* was most stable in different tissues of *Q*. *mongolica* and *Quercus suber* [23], another plants of genus *Quercus*, but its expression level varied significantly under various abiotic environments in our study. These results indicate that the assessment of the expression stability of the reference gene is indispensable under specific experimental conditions before analyzing the expression of the target gene.

Generally, some reference genes can be applied under a variety of experimental environments and/or tissues owing to relatively constant expression level in a specific plant [41–43]. *CYP18* represents a good choice as reference gene. In our study, *CYP18* was regarded as a reliable reference gene in various tissue samples under various abiotic stress environments. *CYP18* has been determined as a stable reference gene for normalizing the gene expression data of different species. For instance, *CYP18* is extensively applied as a reference gene with high stability in a variety of species under various stress environments [43–45]. In this study, *CYP18* was found to be the most appropriate for CS, DL, CdS, CdR, and WL samples. Simultaneously, *CYP18* also ranked well and was one of the best reference genes in SR, CL, DS, WS, and all samples. Therefore, *CYP18* could be prioritized for qRT-PCR analysis of *Q*. *mongolica* in various environments.

We chose *P5CS2* and *CMO*, which were the key genes of the osmoprotectant proline and glycine betaine synthesis pathways under stress conditions such as cold, salt, heat, drought, respectively, as the target genes to confirm the stability of the selected reference genes under various stress treatments and a variety of tissues [6, 27, 46, 47]. *P5CS2* and *CMO* upregulated in other plants under most stress conditions [11, 48]. The results showed that the expression levels of the target genes were remarkably different when the most unstable and the most stable reference genes were utilized. The expression patterns of the target genes were similar when the most stable combination or reference genes was employed in the exploration under different stress conditions. However, compared with using the most stable reference gene, the target gene expression pattern changed markedly when the least stable reference gene was applied, revealing that the unsuitable reference genes may result in incorrect expression outcomes for the target genes [49]. This confirmed that the choice of appropriate reference genes is crucial for qRT-PCR analysis.

## Conclusions

In our study, the stability of the expression of 11 candidate reference genes was explored in distinct tissues and under various abiotic stress conditions in *Q*. *mongolica*. According to the of RefFinder results, *CYP18* was considered the optimal reference gene in cold-treated stems, Cd-treated stems, Cd-treated roots, drought-treated leaves, and weak light-treated leaves. *RPS13* ranked the highest in weak light-treated roots, cold-treated roots, and all samples. *PP2A* and *HIS4* were the most stable in drought-treated roots and weak light-treated stems, respectively. *SAND*, *UBQ10*, *TUA*, and *UBC5* were the most appropriate in drought-treated stems and salt, cold, and Cd-treated leaves, respectively. *ACT97* seemed to be the most appropriate in different tissues, salt-treated stems and roots. The data confirmed expression patterns of the different reference genes under various experimental environments, and we focused on the significance of choosing appropriate reference genes for qRT-PCR analysis in *Q*. *mongolica*. These outcomes will provide a significant basis for investigating the molecular mechanisms underlying the abiotic stress response of *Q*. *mongolica*.

## Supporting information

S1 Fig Specific PCR amplification products of 11 reference genes. M, DL2000 DNA marker. (TIF)

S2 Fig Melting curves for the 11 candidate reference genes in *Q*. *mongolica* by qRT-PCR. (TIF)

S3 Fig Standard curves of 11 candidate reference genes. (PDF)

S1 Table The annotation and comparison information of the 11 reference genes. (XLSX)

S2 Table Gene expression stability (M) of candidate reference genes calculated by geNorm. (DOCX)

S3 Table Pairwise variation (V_n/n+1_) analysis of eleven candidate reference genes calculated by geNorm. (DOCX)
