# Supplementary figures and images for: Selection and validation of reference genes for quantitative real-time PCR of Quercus mongolica Fisch. ex Ledeb under abiotic stresses
Source: PLoS One. 2022 Apr 28;17(4):e0267126. doi: 10.1371/journal.pone.0267126 (PMC9049516; doi:10.1371/journal.pone.0267126)

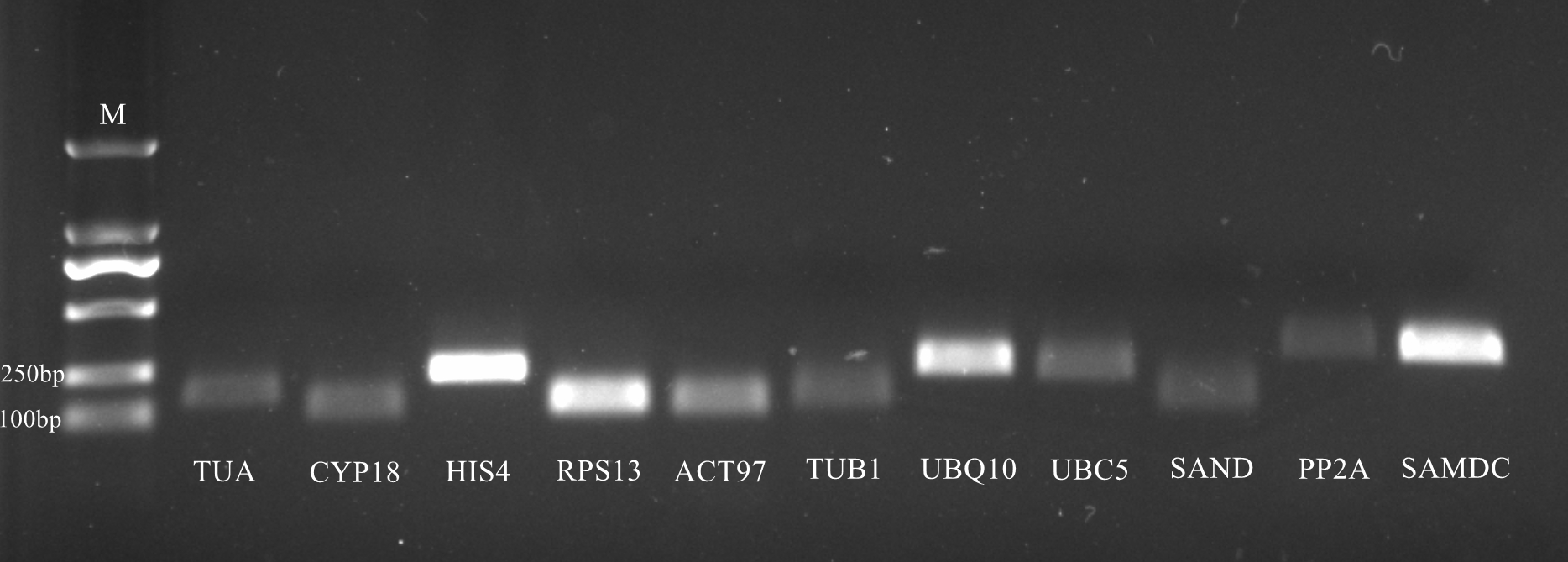

Supplement: S1 Fig — (TIF) [file pone.0267126.s001.tif]

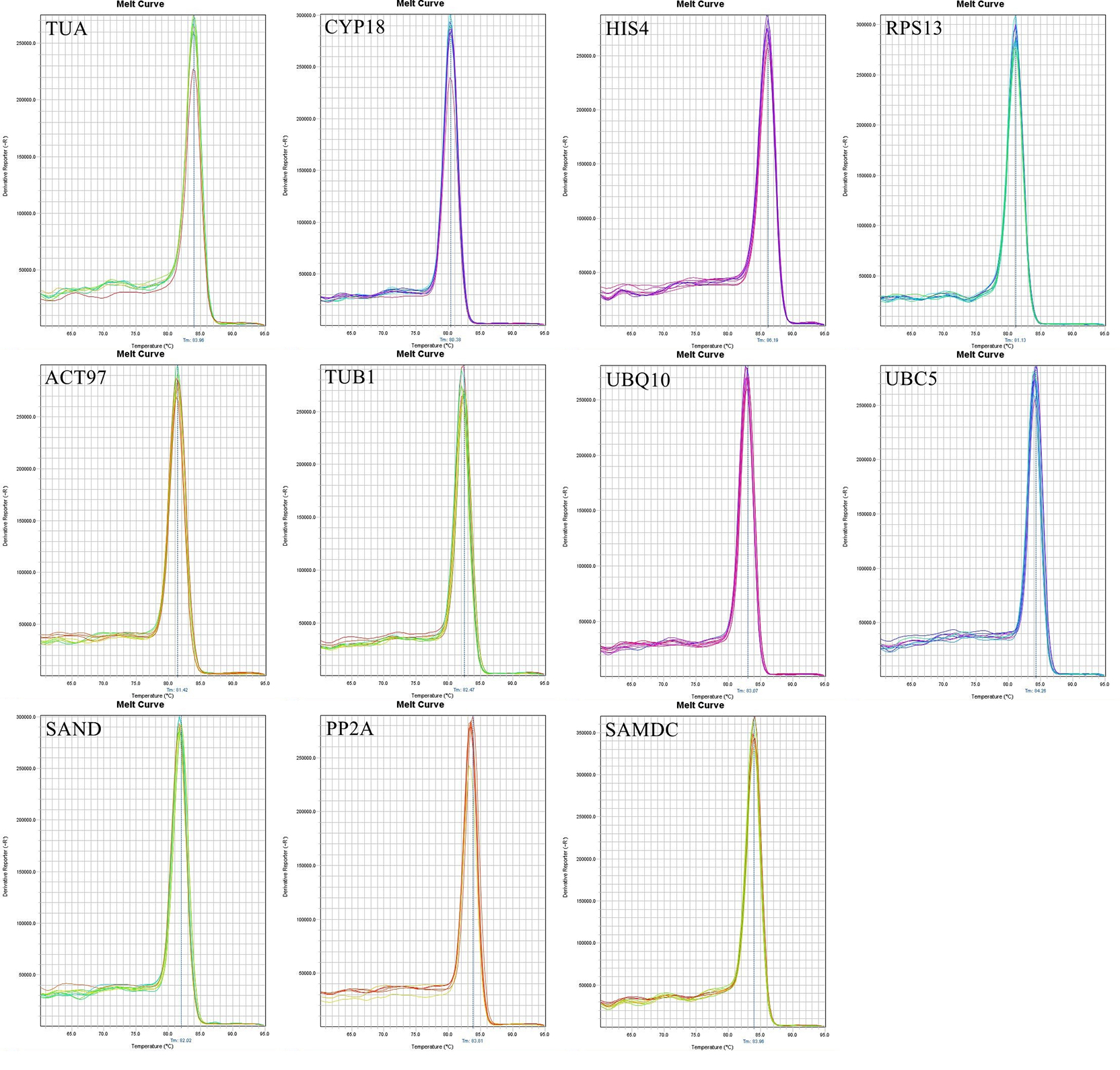

Supplement: S2 Fig — (TIF) [file pone.0267126.s002.tif]

TUA

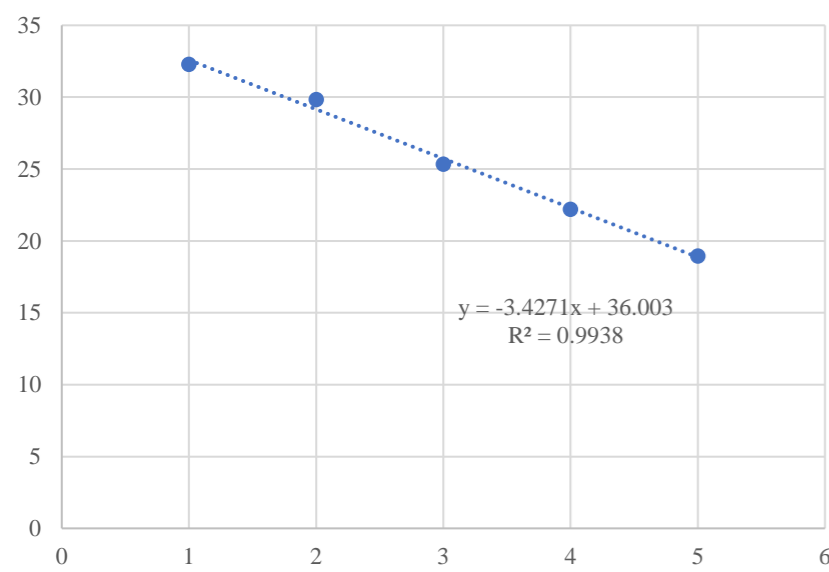

CYP18

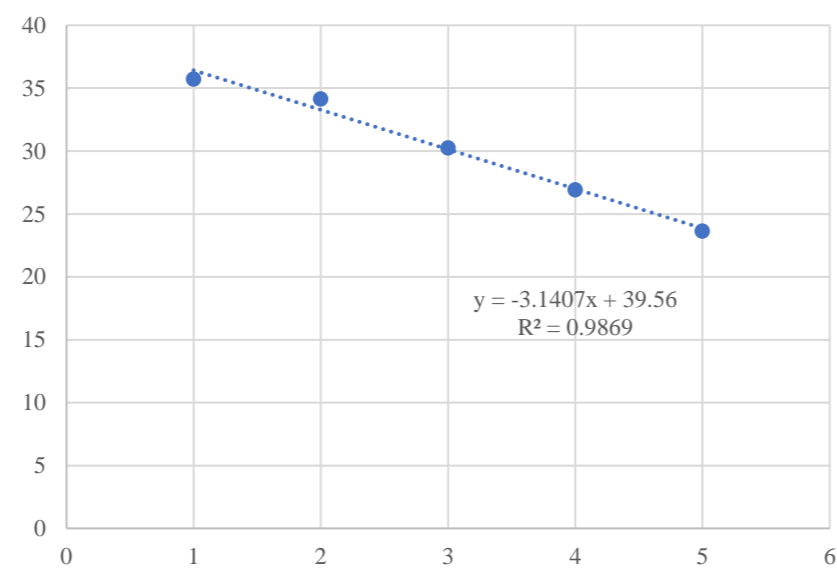

HIS4

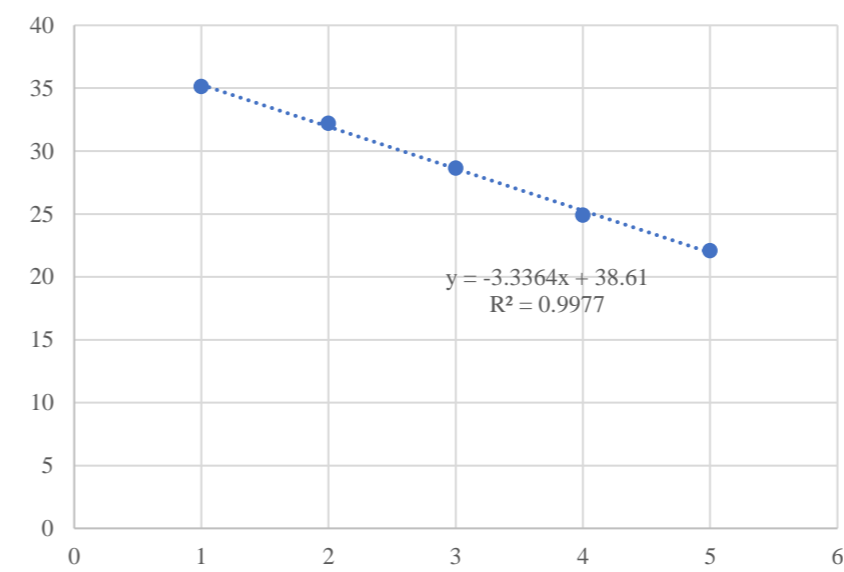

RPS13

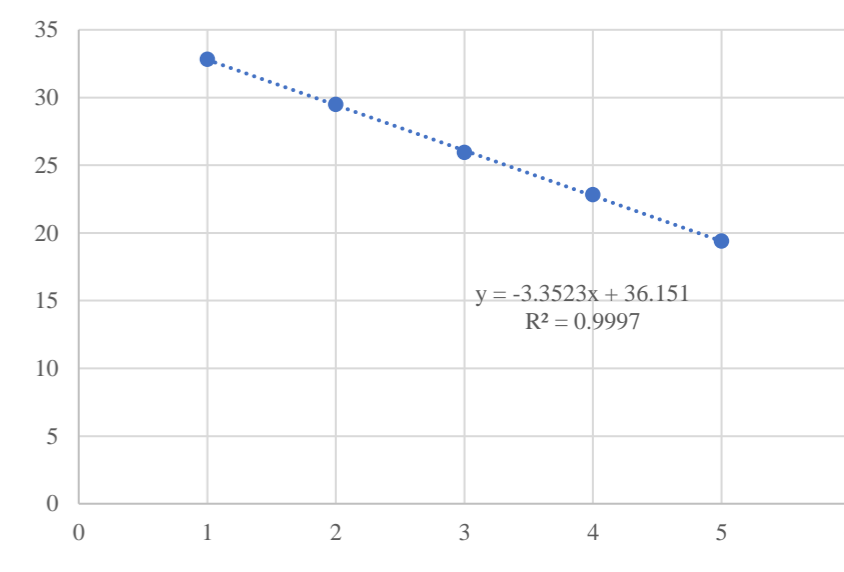

ACT97

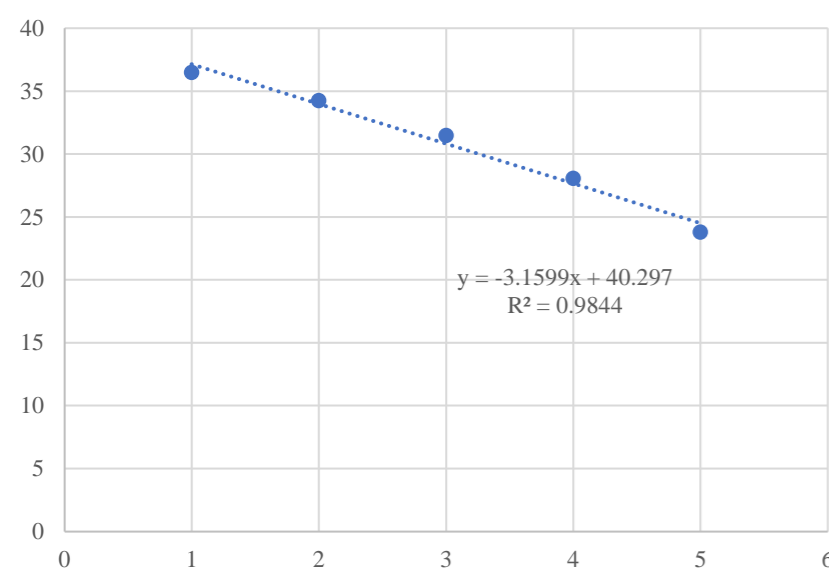

TUB1

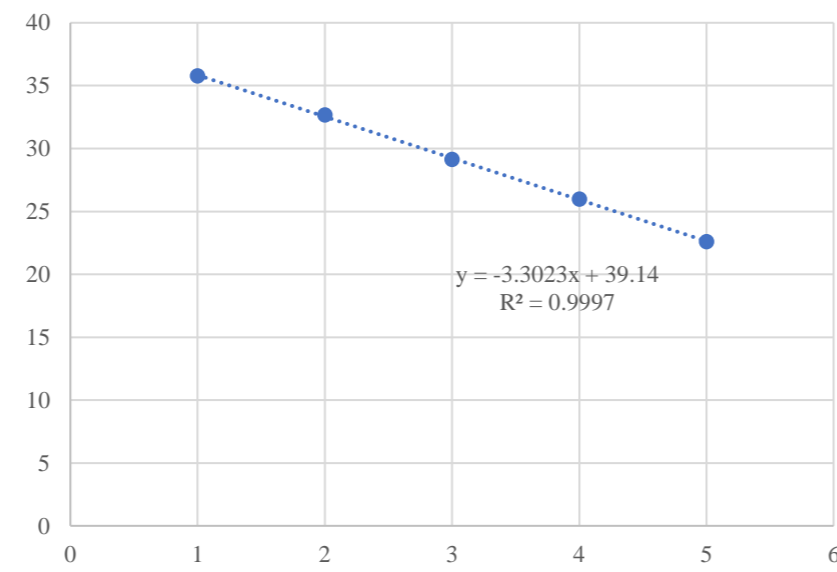

UBQ10

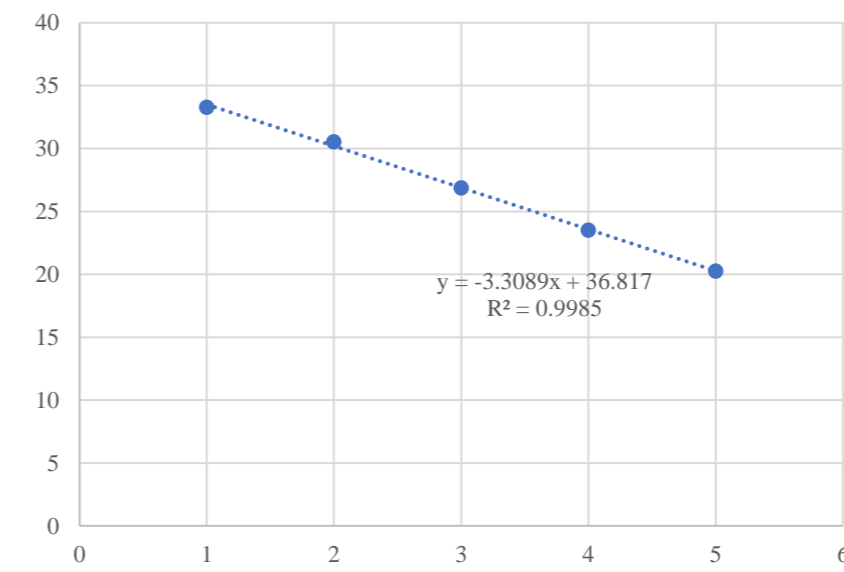

UBC5

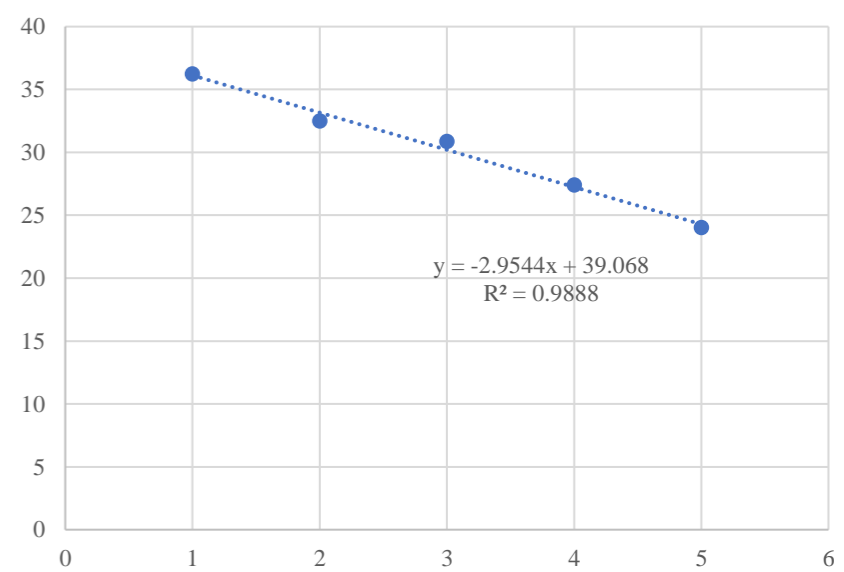

SAND

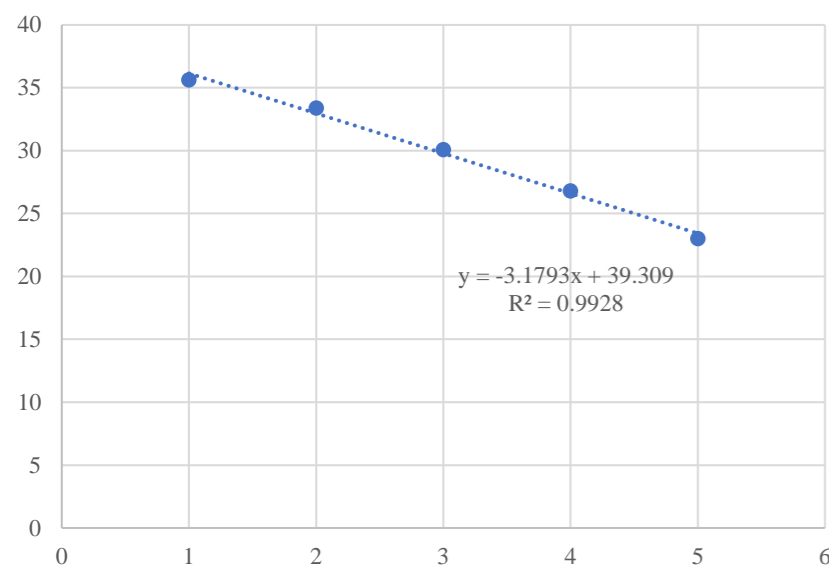

PP2A

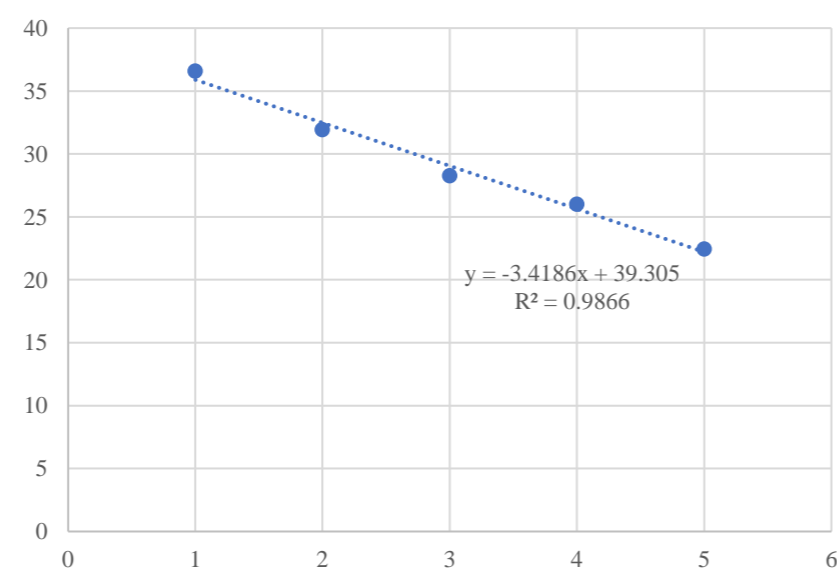

SAMDC

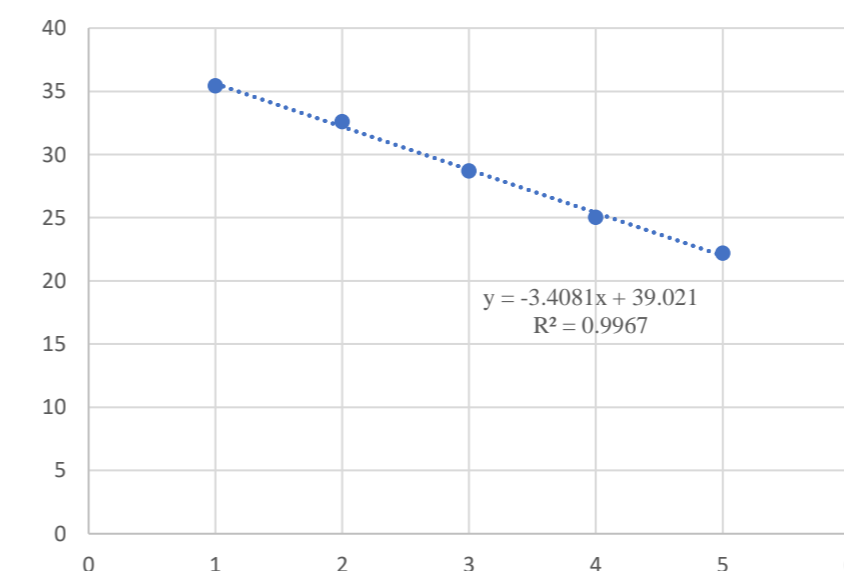

P5CS2

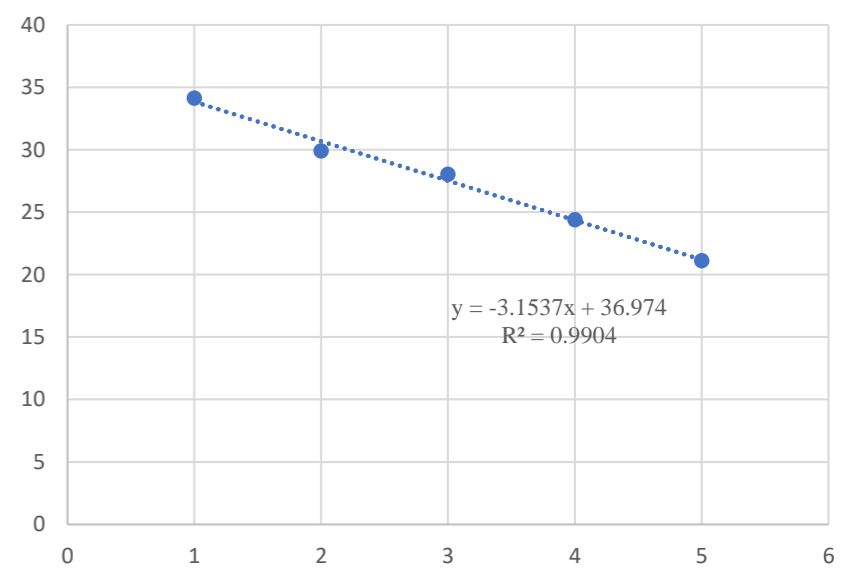

CMO

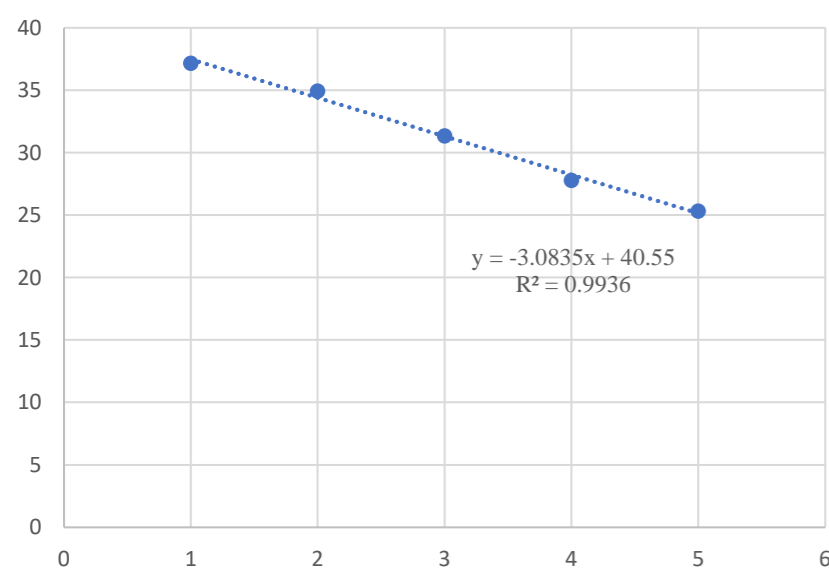

Supplement: S3 Fig — (PDF) [file pone.0267126.s003.pdf]
