# Supplementary material for: Selection and validation of reference genes for quantitative real-time PCR of Quercus mongolica Fisch. ex Ledeb under abiotic stresses
Source: PLoS One. 2022 Apr 28;17(4):e0267126. doi: 10.1371/journal.pone.0267126 (PMC9049516; doi:10.1371/journal.pone.0267126)
Supplement: S2 Table — (DOCX) [file pone.0267126.s005.docx]

**S2 Table.** **Gene expression stability (M) of candidate reference genes calculated by geNorm.**

| **Rank** | **Total** | | **DT** | | **SL** | | **SS** | | **SR** | | **CL** | | **CS** | | **CR** | | **DL** | |
| --- | --- | --- | --- | --- | --- | --- | --- | --- | --- | --- | --- | --- | --- | --- | --- | --- | --- | --- |
|  | **Gene** | **M-value** | **Gene** | **M-value** | **Gene** | **M-value** | **Gene** | **M-value** | **Gene** | **M-value** | **Gene** | **M-value** | **Gene** | **M-value** | **Gene** | **M-value** | **Gene** | **M-value** |
| 1 | CYP18 | 0.748 | HIS4 | 0.110 | CYP18 | 0.379 | UBC5 | 0.591 | CYP18 | 0.469 | ACT97 | 0.169 | HIS4 | 0.210 | ACT97 | 0.559 | UBC5 | 0.343 |
| 2 | RPS13 | 0.748 | TUB1 | 0.110 | TUB1 | 0.379 | SAND | 0.591 | RPS13 | 0.469 | SAND | 0.169 | TUB1 | 0.210 | TUB1 | 0.559 | PP2A | 0.343 |
| 3 | ACT97 | 1.051 | ACT97 | 0.161 | TUA | 0.461 | ACT97 | 0.639 | ACT97 | 0.569 | TUA | 0.284 | ACT97 | 0.292 | CYP18 | 0.608 | RPS13 | 0.414 |
| 4 | TUA | 1.147 | SAND | 0.188 | RPS13 | 0.588 | CYP18 | 0.678 | HIS4 | 0.641 | CYP18 | 0.318 | TUA | 0.565 | RPS13 | 0.773 | HIS4 | 0.516 |
| 5 | HIS4 | 1.269 | UBQ10 | 0.262 | HIS4 | 0.629 | SAMDC | 0.847 | TUA | 0.704 | HIS4 | 0.381 | CYP18 | 0.650 | SAND | 0.839 | CYP18 | 0.686 |
| 6 | TUB1 | 1.345 | CYP18 | 0.324 | UBQ10 | 0.704 | HIS4 | 0.903 | UBC5 | 0.835 | RPS13 | 0.492 | RPS13 | 0.731 | TUA | 1.042 | TUB1 | 0.824 |
| 7 | SAND | 1.429 | PP2A | 0.484 | SAND | 0.769 | TUB1 | 0.952 | SAND | 0.886 | UBC5 | 0.597 | SAND | 0.877 | HIS4 | 1.386 | ACT97 | 0.875 |
| 8 | UBC5 | 1.584 | SAMDC | 0.605 | UBC5 | 0.937 | RPS13 | 1.004 | SAMDC | 0.992 | PP2A | 0.676 | PP2A | 1.005 | UBC5 | 1.693 | SAND | 0.919 |
| 9 | UBQ10 | 1.689 | UBC5 | 0.738 | ACT97 | 1.049 | TUA | 1.062 | PP2A | 1.084 | SAMDC | 0.760 | UBC5 | 1.069 | PP2A | 2.081 | TUA | 0.959 |
| 10 | PP2A | 1.772 | RPS13 | 0.846 | PP2A | 1.115 | PP2A | 1.133 | TUB1 | 1.185 | UBQ10 | 0.833 | SAMDC | 1.155 | UBQ10 | 2.339 | UBQ10 | 1.028 |
| 11 | SAMDC | 2.091 | TUA | 0.998 | SAMDC | 1.206 | UBQ10 | 1.295 | UBQ10 | 1.315 | TUB1 | 0.942 | UBQ10 | 1.245 | SAMDC | 2.594 | SAMDC | 1.670 |
| **Rank** | **DS** | | **DR** | | **CdL** | | **CdS** | | **CdR** | | **WL** | | **WS** | | **WR** | |  | |
|  | **Gene** | **M-value** | **Gene** | **M-value** | **Gene** | **M-value** | **Gene** | **M-value** | **Gene** | **M-value** | **Gene** | **M-value** | **Gene** | **M-value** | **Gene** | **M-value** |  |  |
| 1 | TUA | 0.248 | UBC5 | 0.110 | HIS4 | 0.121 | CYP18 | 0.331 | CYP18 | 0.233 | TUA | 0.111 | ACT97 | 0.071 | CYP18 | 0.428 |  |  |
| 2 | TUB1 | 0.248 | PP2A | 0.110 | SAND | 0.121 | SAND | 0.331 | ACT97 | 0.233 | CYP18 | 0.111 | TUB1 | 0.071 | RPS13 | 0.428 |  |  |
| 3 | HIS4 | 0.343 | SAND | 0.234 | CYP18 | 0.217 | UBC5 | 0.494 | RPS13 | 0.371 | UBQ10 | 0.402 | TUA | 0.449 | TUB1 | 0.596 |  |  |
| 4 | SAND | 0.520 | ACT97 | 0.375 | TUB1 | 0.329 | PP2A | 0.515 | SAND | 0.501 | HIS4 | 0.566 | CYP18 | 0.562 | TUA | 0.635 |  |  |
| 5 | CYP18 | 0.613 | RPS13 | 0.440 | PP2A | 0.379 | TUA | 0.538 | UBC5 | 0.653 | RPS13 | 0.676 | HIS4 | 0.660 | ACT97 | 0.690 |  |  |
| 6 | UBQ10 | 0.716 | CYP18 | 0.521 | UBC5 | 0.493 | HIS4 | 0.570 | TUA | 0.725 | SAND | 0.767 | RPS13 | 0.735 | SAND | 0.850 |  |  |
| 7 | ACT97 | 0.768 | TUA | 0.590 | TUA | 0.586 | TUB1 | 0.610 | SAMDC | 0.922 | UBC5 | 0.897 | UBQ10 | 0.838 | SAMDC | 0.996 |  |  |
| 8 | RPS13 | 0.850 | HIS4 | 0.644 | RPS13 | 0.648 | ACT97 | 0.682 | TUB1 | 1.047 | ACT97 | 1.027 | SAND | 0.898 | PP2A | 1.131 |  |  |
| 9 | PP2A | 0.924 | TUB1 | 0.685 | SAMDC | 0.766 | RPS13 | 0.796 | PP2A | 1.158 | PP2A | 1.153 | UBC5 | 1.142 | UBQ10 | 1.244 |  |  |
| 10 | UBC5 | 0.982 | UBQ10 | 0.777 | UBQ10 | 0.837 | UBQ10 | 0.893 | HIS4 | 1.269 | SAMDC | 1.277 | PP2A | 1.286 | UBC5 | 1.464 |  |  |
| 11 | SAMDC | 1.423 | SAMDC | 1.693 | ACT97 | 0.901 | SAMDC | 0.948 | UBQ10 | 1.402 | TUB1 | 1.438 | SAMDC | 1.546 | HIS4 | 1.720 |  |  |

M-value: expression stability value
