# Supplementary material for: Selection and validation of reference genes for quantitative real-time PCR of Quercus mongolica Fisch. ex Ledeb under abiotic stresses
Source: PLoS One. 2022 Apr 28;17(4):e0267126. doi: 10.1371/journal.pone.0267126 (PMC9049516; doi:10.1371/journal.pone.0267126)
Supplement: S3 Table — (DOCX) [file pone.0267126.s006.docx]

**S3 Table. Pairwise variation (V_n/n+1_ ) analysis of eleven candidate reference genes calculated by geNorm.**

| **Treatment** | **Pairwise variation (V)** | | | | | | | | |
| --- | --- | --- | --- | --- | --- | --- | --- | --- | --- |
|  | **V_2/3_** | **V_3/4_** | **V_4/5_** | **V_5/6_** | **V_6/7_** | **V_7/8_** | **V_8/9_** | **V_9/10_** | **V_10/11_** |
| Total | 0.382 | 0.270 | 0.255 | 0.210 | 0.206 | 0.229 | 0.199 | 0.183 | 0.301 |
| Different tissues | 0.062 | 0.049 | 0.073 | 0.070 | 0.123 | 0.114 | 0.130 | 0.124 | 0.149 |
| Salt treated leaves | 0.155 | 0.166 | 0.121 | 0.131 | 0.125 | 0.174 | 0.149 | 0.128 | 0.132 |
| Salt treated stem | 0.198 | 0.156 | 0.209 | 0.153 | 0.135 | 0.127 | 0.124 | 0.127 | 0.173 |
| Salt treated roots | 0.194 | 0.163 | 0.140 | 0.170 | 0.133 | 0.148 | 0.147 | 0.146 | 0.163 |
| Cold treated leaves | 0.111 | 0.082 | 0.089 | 0.112 | 0.119 | 0.103 | 0.108 | 0.103 | 0.125 |
| Cold treated stem | 0.106 | 0.208 | 0.147 | 0.130 | 0.170 | 0.169 | 0.140 | 0.135 | 0.136 |
| Cold treated roots | 0.189 | 0.225 | 0.167 | 0.230 | 0.309 | 0.310 | 0.363 | 0.313 | 0.322 |
| Drought treated leaves | 0.138 | 0.145 | 0.181 | 0.180 | 0.129 | 0.107 | 0.113 | 0.120 | 0.410 |
| Drought treated stem | 0.124 | 0.168 | 0.137 | 0.140 | 0.116 | 0.128 | 0.116 | 0.109 | 0.305 |
| Drought treated roots | 0.099 | 0.126 | 0.095 | 0.107 | 0.098 | 0.093 | 0.081 | 0.107 | 0.527 |
| Cd treated leaves | 0.086 | 0.106 | 0.081 | 0.116 | 0.108 | 0.092 | 0.125 | 0.101 | 0.097 |
| Cd treated stem | 0.187 | 0.114 | 0.100 | 0.098 | 0.092 | 0.105 | 0.126 | 0.121 | 0.101 |
| Cd treated roots | 0.143 | 0.149 | 0.166 | 0.128 | 0.195 | 0.163 | 0.163 | 0.155 | 0.172 |
| weak light treated leaves | 0.182 | 0.180 | 0.153 | 0.143 | 0.164 | 0.169 | 0.167 | 0.163 | 0.187 |
| weak light treated stem | 0.213 | 0.165 | 0.148 | 0.127 | 0.146 | 0.124 | 0.217 | 0.182 | 0.232 |
| weak light treated roots | 0.216 | 0.146 | 0.145 | 0.187 | 0.181 | 0.180 | 0.166 | 0.225 | 0.246 |
